# Crystal structure and Hirshfeld surface analysis of (*E*)-*N*′-benzyl­idene-4-chloro­benzene­sulfono­hydrazide and of its (*E*)-4-chloro-*N*′-(*ortho*- and *para*-methyl­benzyl­idene)benzene­sulfono­hydrazide derivatives

**DOI:** 10.1107/S2056989018014500

**Published:** 2018-10-19

**Authors:** Akshatha R. Salian, Sabine Foro, B. Thimme Gowda

**Affiliations:** a Department of Chemistry, Mangalore University, Mangalagangotri 574 199, India; b Institute of Materials Science, Darmstadt University of Technology, Alarich-Weiss-Strasse 2, D-64287, Darmstadt, Germany; c Karnataka State Rural Development and Panchayat Raj University, Gadag 582 101, India

**Keywords:** crystal structure, benzyl­idene, benzene­sulfono­hydrazide, *ortho*- and *para*-methyl-substituted derivatives, N—H⋯O hydrogen bonding, C—Cl⋯π inter­actions, Hirshfeld surface analysis, fingerprint plots

## Abstract

(*E*)-*N*′-Benzyl­idene-4-chloro­benzene­sulfono­hydrazide, C_13_H_11_ClN_2_O_2_S, (I), and its *ortho*- and *para*-methyl­substituted derivatives, C_14_H_13_ClN_2_O_2_S, namely (*E*)-4-chloro-*N*′-(2-methyl­benzyl­idene)benzene­sulfono­hydrazide, (II), and (*E*)-4-chloro-*N*′-(4-methyl­benzyl­idene)benzene­sulfono­hydrazide, (III), have been synthesized, characterized spectroscopically and their crystal structures determined to investigate the effect of the substitution site of the benzyl­idene group on the structural and supra­molecular features in these compounds. Compounds (I) and (II) are isotypic while compound (III) is different. All three mol­ecules are bent at the S atom with C—S—N—N torsion angles of −66.0 (3), −66.0 (3) and −58.4 (2)° for (I), (II) and (III), respectively. The hydrazone portions of the mol­ecules, S—N—N=C, are slightly twisted from planarity, with a torsion angle of 166.5 (3)° in (I), 165.4 (3)° in (II) and 157.9 (2)° in (III). The two aromatic rings present in the compounds are inclined to each other by 78.4 (2), 74.8 (2) and 76.9 (1)° in (I), (II) and (III), respectively. In the crystal structure of the parent compound (I), and of the *ortho*-methyl derivative (II), an N—H⋯O hydrogen bond links the mol­ecules into chains along [001], which are inter­connected by weak inter­molecular C—H⋯O inter­actions, generating layers lying parallel to the *bc* plane. In the crystal of the *para* derivative (III), however, the packing is significantly different. Here mol­ecules are linked by pairs of N—H⋯O hydrogen bonds, forming inversion dimers with an *R*_2_^2^(8) ring motif. The dimers are then linked by C—Cl⋯π inter­actions, forming ribbons propagating along [10]. Hirshfeld surface analyses show that the van der Waals inter­actions constitute the major contribution to the inter­molecular inter­actions in the crystal structures of all three compounds. The fingerprint plots indicate that the H⋯H contacts make the largest contributions to the Hirshfeld surfaces.

## Abstract 2

The crystal structures of (*E*)-*N*′-(benzyl­idene)-4-chloro­benzene­sulfono­hydrazide (I) and its *ortho*- and *para*-methyl-substituted benzyl­idene derivatives, (*E*)-*N*′-(2-methyl­benzyl­idene)-4-chloro-benzene­sulfono­hydrazide (II) and (*E*)-*N*′-(4-methyl­benzyl­idene)-4-chloro­benzene­sulfono­hydrazide (III), have been studied to investigate the effect of substitution on the structural and supra­molecular features of these compounds.

## Chemical context   

Schiff bases are an important class of compounds in the field of coordination chemistry and catalysis (Mahfouz *et al.*, 2015 ▸). The photochromic and thermochromic properties of Schiff bases make their study inter­esting (Girisha *et al.*, 2018 ▸). They form second-order NLO organic materials, which are being used in computers, optical communication and medical imaging (Zarei *et al.*, 2015 ▸). Hydrazones also play an important role in curing diseases effectively with less toxicity. Sulfonyl hydrazones are known for their good enzymatic modulation, analgesic, anti-Alzheimer’s, anti­depressant and anti­diabetic activities (Cunha *et al.*, 2016 ▸). To investigate the impact of substitution, and also the variation of the site of substituent, on the structural parameters and the hydrogen-bonding inter­actions, we report herein on the synthesis and crystal structures of (*E*)-*N*′-(benzyl­idene)-4-chloro­benzene­sulfono­hydrazide (I) and its *ortho-* and *para-*methyl­substituted benzyl­idene derivatives, (II) and (III), respectively.
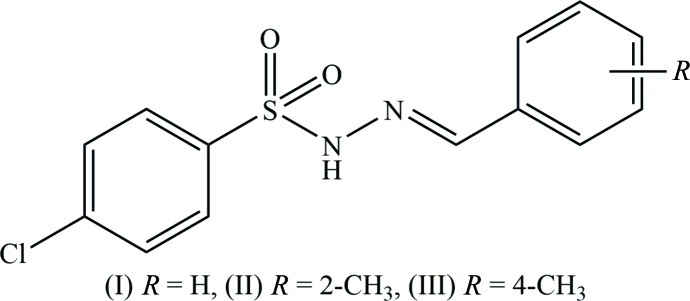


## Structural commentary   

**Figure 1:**
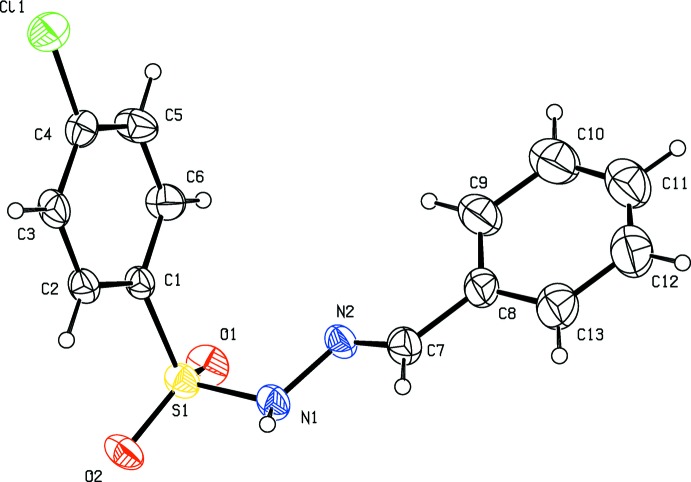
Mol­ecular structure of (I), showing the atom labelling and displacement ellipsoids drawn at the 30% probability level.

The title hydrazide (I) and its derivatives, (II) and (III), crystallize in the monoclinic crystal system with space group *P2**_1_**/c* for (I) and (II) and *P2**_1_**/n* for (III). The mol­ecular structures of compounds (I), (II) and (III) are illustrated in Figs. 1 ▸, 2 ▸ and 3 ▸, respectively. All three mol­ecules adopt an *E* configuration about the C=N bond of the central imine group. In the *ortho*-methyl-substituted derivative (II), the N—H and C—H bonds in the hydrazide part are *anti* with respect to the methyl substituent. These parts of the mol­ecules, S—N—N=C, show similar bond lengths of 1.258 (5), 1.272 (5) and 1.273 (3) Å for C7=N2 and 1.394 (5), 1.407 (5) and 1.393 (2) Å for N1—N2 in compounds (I), (II) and (III), respectively. These bond lengths are consistent with the C=N double-bond and N—N single-bond lengths, respectively. Furthermore, the S—N—N=C segments are slightly twisted from planarity, with torsion angles of 166.5 (3)° in (I), 165.4 (3)° in (II) and 157.9 (2)° in (III). All three compounds are bent at the S atom with C—S—N—N torsion angles of −66.0 (3), −66.0 (3) and −58.4 (2)° for (I), (II) and (III), respectively. The two aromatic rings present in these compounds are inclined to each other by 78.4 (2), 74.8 (2) and 76.9 (1)° in (I), (II) and (III), respectively. Hence the conformations of (I) and (II) are very similar while that of (III) is slightly different.

## Supra­molecular features   

**Figure 4:**
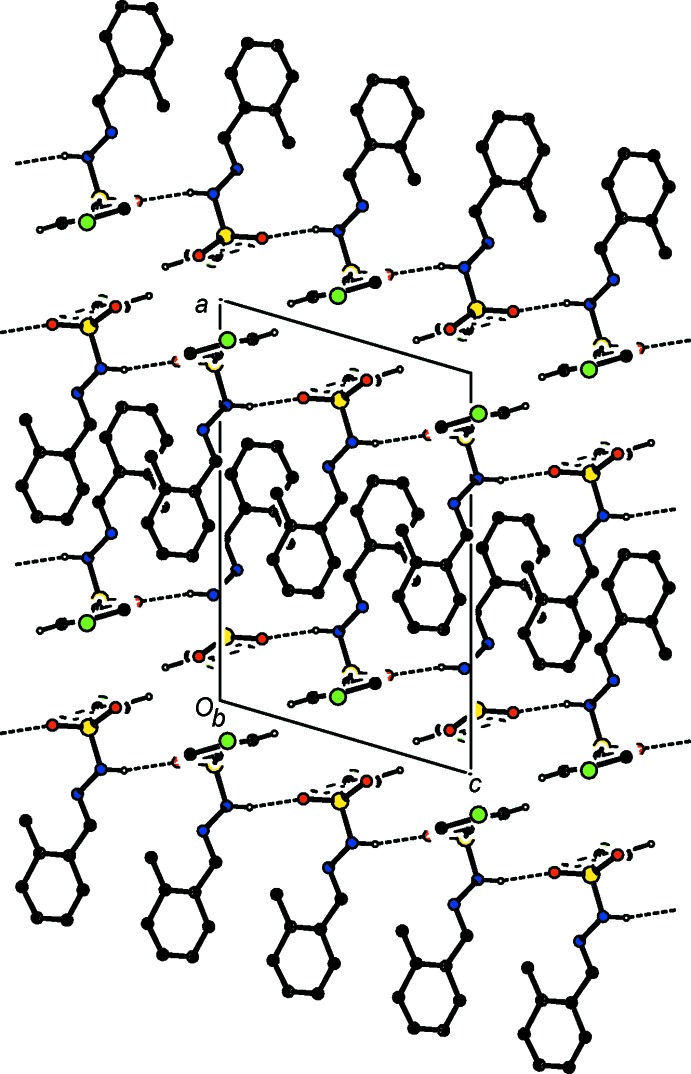
A partial view along the *b* axis of the crystal packing of (II), with hydrogen bonds shown as dashed lines. Only the H atoms involved in the intermolecular interactions have been included.

**Figure 5:**
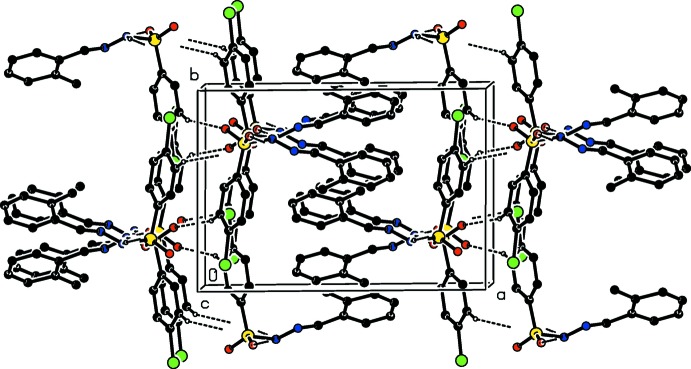
A view along the *c* axis of the crystal packing of (II), with hydrogen bonds shown as dashed lines. Only the H atoms involved in the intermolecular interactions have been included.

**Figure 6:**
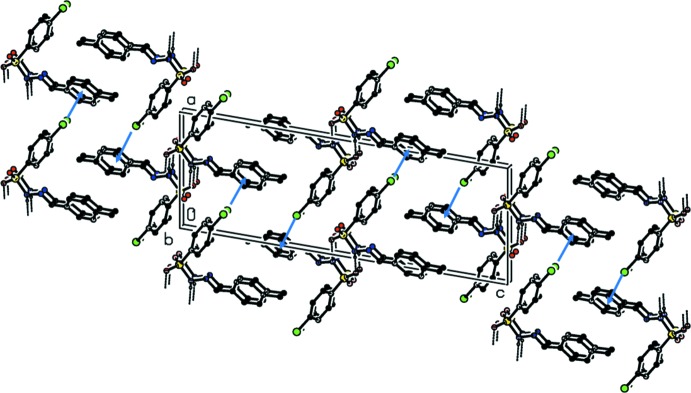
A view along the *b* axis of the crystal packing of (III), with hydrogen bonds shown as dashed lines. Only the H atoms involved in the intermolecular interactions have been included. The C—Cl⋯π interactions are indicated by blue arrows.

In the crystals of all three compounds, an O atom of the sulfonyl group acts as an acceptor and the amino H atom of the hydrazide segment as a donor in N—H⋯O hydrogen-bonding inter­actions with neighbouring mol­ecules (Tables 1 ▸, 2 ▸ and 3 ▸). The patterns of the hydrogen-bonding inter­actions in the crystal structures of (I) and (II) are very similar, and will be illustrated for compound (II) only. The N—H⋯O hydrogen-bonding inter­actions result in a *C*(4) graph-set motif generating chains propagating along the *c*-axis direction (Fig. 4 ▸). These chains are linked by weak C—H⋯O inter­actions involving an aromatic H atom of the benzyl­idenephenyl ring and a sulfonyl O atom, resulting in the formation of layers lying parallel to the *bc* plane (Tables 2 ▸ and 3 ▸, and Fig. 5 ▸). On changing the position of the methyl substituent from *ortho*- to *para*- the crystal packing changes significantly. Mol­ecules are now linked by pairs of N—H⋯O hydrogen bonds, forming inversion dimers enclosing 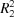
(8) loops (Fig. 6 ▸, Table 3 ▸). The dimers are linked by a C—Cl⋯π inter­action, forming ribbons that propagate along the [1
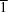
0] direction (Fig. 6 ▸, Table 3 ▸).

## Hirshfeld Surface analysis   

**Figure 7:**
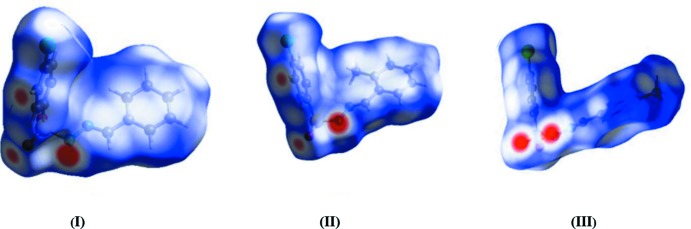
Hirshfeld surface mapped over *d*_norm_ for (I), (II) and (III).

The nature of the inter­molecular contacts and their qu­anti­tative contributions to the crystal packing in all the three title compounds were analysed by Hirshfeld surface analysis and two-dimensional fingerprint plots, generated using *CrystalExplorer3.1* (McKinnon *et al.*, 2004 ▸; Spackman & Jayatilaka, 2009 ▸; Wolff *et al.*, 2012 ▸). The Hirshfeld surfaces of the three compounds mapped over *d*_norm_ are shown in Fig. 7 ▸. The N—H⋯O inter­actions between the corresponding donor and acceptor atoms are visualized as bright-red spots and represent the short inter­atomic inter­actions in the crystal structures. The presence of two other light-red spots in (I) and (II) correspond to the C—H⋯O inter­actions, which are considered to be weak inter­actions.

**Figure 8:**
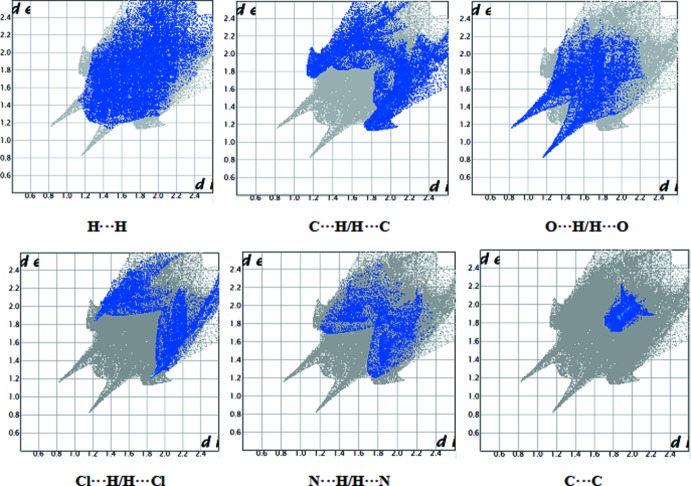
Two-dimensional fingerprint plots for (I), showing the contributions of different types of inter­actions.

The two-dimensional fingerprint plots for the contacts H⋯H, C⋯H/H⋯C, O⋯H/H⋯O, Cl⋯H/H⋯Cl, C⋯C and N⋯H/H⋯N are illustrated in Figs. 8 ▸ and 9 ▸, for (I) and (III), respectively. The fingerprint plots of various contacts and their percentage contribution to the Hirshfeld surfaces are similar in (I) and (II) but, as expected, different from those for (III) (see Table 4 ▸). H⋯H contacts are the major contributors to the Hirshfeld surface: 30.1% in (I), 34.0% (II) and 38.0% in (III). The C⋯H/H⋯C contacts make the second largest contribution, *i.e*. 22.7, 20.2 and 18.0% for (I), (II) and (III), respectively. This is followed by O⋯H/H⋯O contacts arising from N—H⋯O and C—H⋯O inter­actions, contributing 16.1% in (I) and (II), and 15.7% in (III). N⋯H/H⋯N contacts arising from O—H⋯N hydrogen bonds contribute 6.3, 5.2 and 3.9%, respectively, in (I), (II) and (III). Cl⋯H/H⋯Cl inter­actions make a relatively significant contribution to the total Hirshfeld surfaces, comprising 12.1% in (I), 12.3% in (II) and 9.4% in (III). The C⋯C contacts representing π–π inter­actions contribute 5.2, 5.0 and 2.1% in (I), (II) and (III), respectively. Cl⋯O/O⋯Cl contacts comprise 5.0% in (I), 4.8% in (II) and 2.3% in (III). Weak Cl⋯Cl, C⋯O/O⋯C and C⋯S/S⋯C inter­actions are also observed; however, they exhibit minimal respect contributions of 0.5, 1.0 and 0% in (I), 0.5, 1.0, 0.1% in (II) and 0, 2.6 and 0.1% in (III), reflecting negligible or no effect on the mol­ecular packing.

The most significant difference for compounds (I) and (II) compared to compound (III) is the presence of a relatively strong Cl⋯C/C⋯Cl inter­action in (III), in accordance with the C—Cl⋯π inter­action in the crystal (Table 3 ▸), which makes a contribution of 5.3%, while for (I) and (II) this inter­action is not present.

## Database survey   

The crystal structures of (*E*)-*N*′-(4-chloro­benzyl­idene)-4-methyl­benzene­sulfono­hydrazide (IV) (Balaji *et al.*, 2014 ▸) and *N*′-[(*E*)-4-methyl­benzyl­idene]4-methyl­benzene­sulfono­hydra­zide (V) (Tabatabaee *et al.*, 2007 ▸) have been reported. They exhibit an *E* configuration with respect to the C=N bond and an almost perpendicular orientation of the two aromatic rings with dihedral angles of 81.9 (3)° in (IV) and 82.4 (1)° in (V), very similar to the values of 78.4 (2), 74.8 (2) and 76.9 (1)° in (I), (II) and (III), respectively. In the structures of these related compounds (I)–(V) and also those of benzyl­idene, 3,3-di­phenyl­allyl­idene (Mehrabi & Kia, 2009 ▸; Mehrabi *et al.*, 2008 ▸), 4-bromo/5-bromo-2-hy­droxy/5-chloro-2-hy­droxy (Kia *et al.*, 2008*a* ▸,*b* ▸) and 2-hy­droxy-5-iodo (Ghorbanloo & Notash, 2012 ▸) derivatives of *p*-toluene­sulfono­hydrazide, the aryl­sulfono­hydrazide mol­ecules are directly connected to one another *via* significant N—H⋯O hydrogen-bonding inter­actions involving a sulfonyl oxygen atom and the amino hydrogen atom.

## Synthesis and crystallization   

**Synthesis of 4-chloro­benzene­sulfono­hydrazide**

To 4-chlro­benzene­sulfonyl chloride (0.01 mol) dissolved in propanol (30 ml), 99% hydrazine hydrate (5 ml) was added at 273 K under constant stirring. The stirring continued for 15 min at 273 K and then at 303 K for 3 h. After completion of the reaction (monitored by TLC), the reaction mixture was concentrated by evaporating the excess propanol. The solid product, 4-chloro­benzene­sulfono­hydrazide was washed with cold water and dried.

**Synthesis of compounds (I), (II) and (III)**

The parent, *ortho*- and *para*- substituted (*E*)-*N*′-(benzyl­idene)-4-chloro­benzene­sulfono­hydrazides (I), (II) and (III), were synthesized by refluxing mixtures of 4-chloro­benzene­sulfono­hydrazide (0.01 mol) and benzaldehyde, 2-methyl-benzaldehyde or 4-methyl­benzaldehyde (0.01 mol), respect­ively, in ethanol (30 ml) and two drops of glacial acetic acid for 4 h. The reaction mixtures were cooled to room temperature and concentrated by evaporating the excess of solvent. The solid products (I), (II) and (III) obtained were washed with cold water, dried and recrystallized to constant melting points from ethanol to obtain the pure compounds. The purity of the compounds was checked by TLC. Single crystals of the hydrazides suitable for single crystal X-ray diffraction analysis were obtained by slow evaporation of their DMF solutions at room temperature**.** All three compounds were characterized by measuring their IR, ^1^H and ^13^C NMR spectra.

**(** ***E*** **)-** ***N*** **-(benzyl­idene) 4-chloro­benzene­sulfono­hydrazide (I):**

Plate-like colourless single crystals; m.p. 381–382 K; IR (cm^−1^): 3174.8 (N—H asym stretch), 1577.8 (C=N), 1323.2 (S=O asym stretch) and 1159.2 (S=O sym stretch); ^1^H NMR (400 MHz, CDCl_3_, δ ppm): 7.29–7.33 (*m*, 3H, Ar-H), 7.52 (*t*, 2H, Ar-H, *J* = 7.44), 7.53–7.56 (*m*, 3H, Ar-H), 7.94 (*d*, 1H, Ar-H, *J* = 8.4Hz), 7.93 (*s*, 1H), 11.54 (*s*, 1H) and ^13^C NMR (400 MHz, CDCl_3_, δ ppm): 125.46, 127.21, 127.72, 127.86, 128.63, 132.23, 136.52, 136.99, 146.11.

**(** ***E*** **)-** ***N*** **-(2-methyl­benzyl­idene) 4-chloro­benzene­sulfono­hydrazide (II):**

Rod-shaped colourless single crystals; m.p. 399–400 K; IR (cm^−1^): 3155.5 (N—H asym stretch), 1585.6 (C=N), 1325.1 (S=O asym stretch) and 1153.4 (S=O sym stretch); ^1^H NMR (400 MHz, CDCl_3_, δ ppm): 2.33 (*s*, 3H), 7.09–7.17 (*m*, 1H, Ar-H), 7.21–7.26 (*m*, 1H, Ar-H), 7.43–7.48 (*m*, 1H, Ar-H), 7.63 (*d*, 1H, Ar-H, *J* = 7.7 Hz), 7.86 (*d*, 2H, Ar-H, *J* = 8.6 Hz), 7.93 (*d*, 2H, Ar-H, *J* = 8.5 Hz), 8.08 (*s*, 1H), 11.67 (*s*, 1H) and ^13^C NMR (400 MHz, CDCl_3_, δ ppm): 19.77, 126.17, 127.27, 129.13, 129.31, 129.81, 130.23, 131.14, 136.88, 139.72, 140.26, 147.62.

**(** ***E*** **)-** ***N*** **-(4-methyl­benzyl­idene) 4-chloro­benzene­sulfono­hydrazide (III):**

Rod-shaped colourless single crystals; m.p. 425–426K; IR (cm^−1^): 3184.5 (N—H asym stretch), 1580.7 (C=N), 1326.5 (S=O asym stretch) and 1163.3 (S=O sym stretch); ^1^H NMR (400 MHz, CDCl_3_, δ ppm): 2.27 (*s*, 3H), 7.12 (*d*, 2H, Ar-H, *J* = 8.0 Hz), 7.41 (*d*, 2H, Ar-H, *J* = 8.0Hz), 7.52–7.57 (*m*, 2H, Ar-H), 7.86–7.90 (*m*, 2H, Ar-H), 7.92 (*s*, 1H), 11.40 (*s*, 1H) and ^13^C NMR (400 MHz, CDCl_3_, δ ppm): 20.96, 126.64, 128.95, 129.61, 130.75, 137.75, 139.41, 139.78, 145.66.

## Refinement   

Crystal data, data collection and structure refinement details are summarized in Table 5 ▸. For all three compounds, the H atom of the NH group was located in difference-Fourier maps and later restrained to N—H = 0.86 (2) Å. C-bound H atoms were positioned with idealized geometry and refined using a riding model: C—H = 0.93–0.96 Å with *U*_iso_(H) = 1.5*U*_eq_(C-meth­yl) and 1.2*U*_eq_(C-aromatic, N) for other H atoms. The *U^ij^* components of C9, C10, C11 and C12 in (I) and C10, C11, C12 and C13 in (II) and (III) were restrained to approximate isotropic behaviour.

## Supplementary Material

Crystal structure: contains datablock(s) I, II, III, global. DOI: 10.1107/S2056989018014500/su5455sup1.cif

Structure factors: contains datablock(s) I. DOI: 10.1107/S2056989018014500/su5455Isup2.hkl

Structure factors: contains datablock(s) II. DOI: 10.1107/S2056989018014500/su5455IIsup3.hkl

Structure factors: contains datablock(s) III. DOI: 10.1107/S2056989018014500/su5455IIIsup4.hkl

CCDC references: 1578698, 1578700, 1578702

Additional supporting information:  crystallographic information; 3D view; checkCIF report

## Figures and Tables

**Figure 2:**
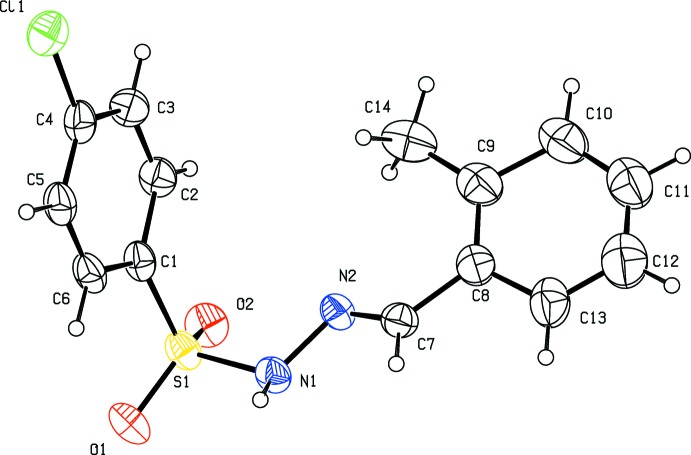
Mol­ecular structure of (II), showing the atom labelling and displacement ellipsoids drawn at the 30% probability level.

**Figure 3:**
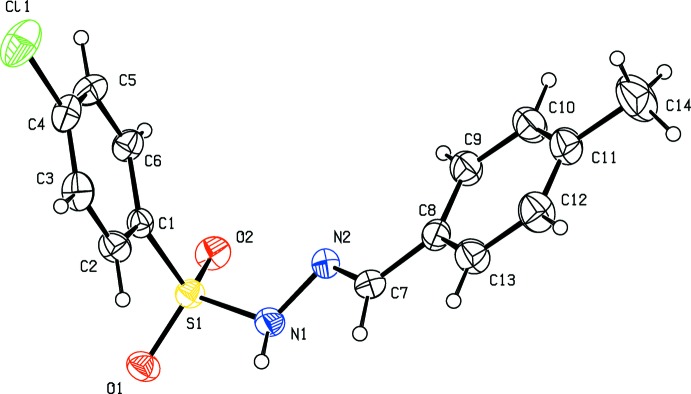
Mol­ecular structure of (III), showing the atom labelling and displacement ellipsoids drawn at the 30% probability level.

**Figure 9:**
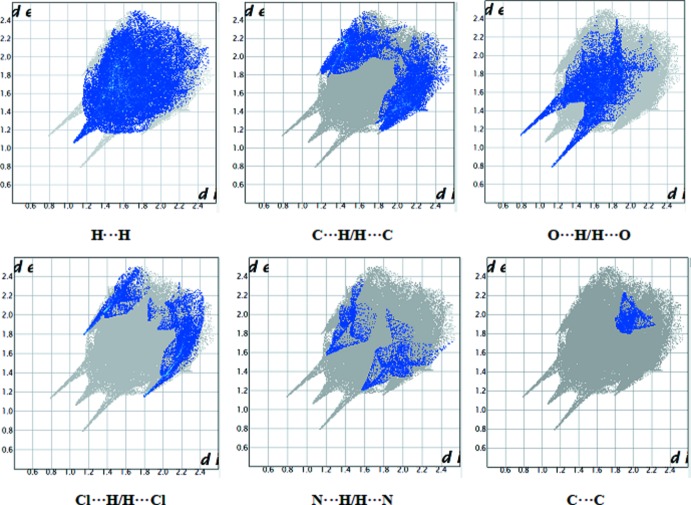
Two-dimensional fingerprint plots for (III), showing the contributions of different types of inter­actions.

**Table 1:** Hydrogen-bond geometry (Å, °) for (I)

*D*—H⋯*A*	*D*—H	H⋯*A*	*D*⋯*A*	*D*—H⋯*A*
N1—H1*N*⋯O1^i^	0.83 (2)	2.14 (3)	2.897 (4)	152 (4)
C3—H3⋯O2^ii^	0.93	2.43	3.305 (5)	158

Symmetry codes: (i) 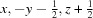; (ii) 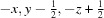.

**Table 2:** Hydrogen-bond geometry (Å, °) for (II)

*D*—H⋯*A*	*D*—H	H⋯*A*	*D*⋯*A*	*D*—H⋯*A*
N1—H1*N*⋯O2^i^	0.86 (2)	2.06 (2)	2.913 (4)	168 (4)
C5—H5⋯O1^ii^	0.93	2.44	3.303 (5)	155

Symmetry codes: (i) 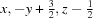; (ii) 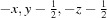.

**Table 3:** Hydrogen-bond geometry (Å, °) for (III) *Cg*1 is the centroid of ring C8-C13.

*D*—H⋯*A*	*D*—H	H⋯*A*	*D*⋯*A*	*D*—H⋯*A*
N1—H1*N*⋯O1^i^	0.85 (2)	2.09 (2)	2.935 (2)	177 (2)
C4—Cl1⋯*Cg*1^ii^	1.74 (1)	3.47 (1)	5.175 (3)	168 (1)

Symmetry codes: (i) 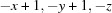; (ii) 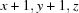.

**Table 4:** Hirshfeld contact inter­actions (%)

Contact type	(I)	(II)	(III)
H⋯H	30.1	34.0	38.0
C⋯H/H⋯C	22.7	20.2	18.0
O⋯H/H⋯O	16.1	16.1	15.7
Cl⋯H/H⋯Cl	12.1	12.3	9.4
N⋯H/H⋯N	6.3	5.2	3.9
C⋯C	5.2	5.0	2.1
Cl⋯C/C⋯Cl	0	0	5.3
Cl⋯O/O⋯Cl	5.0	4.8	2.3
C⋯O/O⋯C	1.0	1.0	2.6
Cl⋯Cl	0.5	0.5	0
C⋯S/S⋯C	0	0.1	0.1

**Table 5:** Experimental details

	(I)	(II)	(III)
Crystal data			
Chemical formula	C_13_H_11_ClN_2_O_2_S	C_14_H_13_ClN_2_O_2_S	C_14_H_13_ClN_2_O_2_S
*M*_r_	294.75	308.77	308.77
Crystal system, space group	Monoclinic, *P*2_1_/*c*	Monoclinic, *P*2_1_/*c*	Monoclinic, *P*2_1_/*n*
Temperature (K)	293	293	293
*a*, *b*, *c* (Å)	14.949 (2), 10.020 (1), 9.641 (1)	15.034 (2), 10.180 (1), 9.8119 (9)	9.406 (1), 5.8353 (6), 26.930 (2)
β (°)	104.27 (1)	106.34 (1)	99.621 (9)
*V* (Å^3^)	1399.6 (3)	1441.0 (3)	1457.3 (2)
*Z*	4	4	4
Radiation type	Mo *K*α	Mo *K*α	Mo *K*α
μ (mm^−1^)	0.42	0.41	0.41
Crystal size (mm)	0.20 × 0.16 × 0.08	0.22 × 0.16 × 0.08	0.48 × 0.16 × 0.14

Data collection			
Diffractometer	Oxford Diffraction Xcalibur diffractometer with Sapphire CCD detector	Oxford Diffraction Xcalibur diffractometer with Sapphire CCD detector	Oxford Diffraction Xcalibur diffractometer with Sapphire CCD detector
Absorption correction	Multi-scan (*CrysAlis RED*; Oxford Diffraction, 2009 ▸)	Multi-scan (*CrysAlis RED*; Oxford Diffraction, 2009 ▸)	Multi-scan (*CrysAlis RED*; Oxford Diffraction, 2009 ▸)
*T*_min_, *T*_max_	0.921, 0.967	0.915, 0.968	0.829, 0.945
No. of measured, independent and observed [*I* > 2σ(*I*)] reflections	4831, 2547, 1034	5157, 2636, 1713	9653, 2652, 2106
*R*_int_	0.075	0.038	0.027
(sin θ/λ)_max_ (Å^−1^)	0.602	0.602	0.602

Refinement			
*R*[*F* ^2^ > 2σ(*F* ^2^)], *wR*(*F* ^2^), *S*	0.062, 0.113, 0.91	0.067, 0.195, 1.07	0.040, 0.095, 1.05
No. of reflections	2547	2636	2652
No. of parameters	175	185	185
No. of restraints	30	32	1
H-atom treatment	H atoms treated by a mixture of independent and constrained refinement	H atoms treated by a mixture of independent and constrained refinement	H atoms treated by a mixture of independent and constrained refinement
Δρ_max_, Δρ_min_ (e Å^−3^)	0.23, −0.21	0.66, −0.32	0.21, −0.31

Computer programs: *CrysAlis CCD* and *CrysAlis RED* (Oxford Diffraction, 2009 ▸), *SHELXS2013* (Sheldrick, 2008 ▸), *SHELXL2014* (Sheldrick, 2015 ▸) and *PLATON* (Spek, 2009 ▸).

## supplementary crystallographic information

### (E)-N'-Benzylidene-4-chlorobenzenesulfonohydrazide (I) Crystal data

**Table:**

C_13_H_11_ClN_2_O_2_S	*F*(000) = 608
*M**_r_* = 294.75	*D*_x_ = 1.399 Mg m^−^^3^
Monoclinic, *P*2_1_/*c*	Mo *K*α radiation, λ = 0.71073 Å
*a* = 14.949 (2) Å	Cell parameters from 692 reflections
*b* = 10.020 (1) Å	θ = 2.8–28.0°
*c* = 9.641 (1) Å	µ = 0.42 mm^−^^1^
β = 104.27 (1)°	*T* = 293 K
*V* = 1399.6 (3) Å^3^	Plate, colourless
*Z* = 4	0.20 × 0.16 × 0.08 mm

### (E)-N'-Benzylidene-4-chlorobenzenesulfonohydrazide (I) Data collection

**Table:**

Oxford Diffraction Xcalibur diffractometer with Sapphire CCD detector	1034 reflections with *I* > 2σ(*I*)
Radiation source: Enhance (Mo) X-ray Source	*R*_int_ = 0.075
Rotation method data acquisition using ω scans.	θ_max_ = 25.4°, θ_min_ = 2.8°
Absorption correction: multi-scan (CrysAlis RED; Oxford Diffraction, 2009)	*h* = −16→18
*T*_min_ = 0.921, *T*_max_ = 0.967	*k* = −12→8
4831 measured reflections	*l* = −8→11
2547 independent reflections	

### (E)-N'-Benzylidene-4-chlorobenzenesulfonohydrazide (I) Refinement

**Table:**

Refinement on *F*^2^	30 restraints
Least-squares matrix: full	Hydrogen site location: mixed
*R*[*F*^2^ > 2σ(*F*^2^)] = 0.062	H atoms treated by a mixture of independent and constrained refinement
*wR*(*F*^2^) = 0.113	*w* = 1/[σ^2^(*F*_o_^2^) + (0.0368*P*)^2^] where *P* = (*F*_o_^2^ + 2*F*_c_^2^)/3
*S* = 0.91	(Δ/σ)_max_ < 0.001
2547 reflections	Δρ_max_ = 0.23 e Å^−^^3^
175 parameters	Δρ_min_ = −0.21 e Å^−^^3^

### (E)-N'-Benzylidene-4-chlorobenzenesulfonohydrazide (I) Special details

**Table:**

Geometry. All esds (except the esd in the dihedral angle between two l.s. planes) are estimated using the full covariance matrix. The cell esds are taken into account individually in the estimation of esds in distances, angles and torsion angles; correlations between esds in cell parameters are only used when they are defined by crystal symmetry. An approximate (isotropic) treatment of cell esds is used for estimating esds involving l.s. planes.

### (E)-N'-Benzylidene-4-chlorobenzenesulfonohydrazide (I) Fractional atomic coordinates and isotropic or equivalent isotropic displacement parameters (Å^2^)

**Table:**

	*x*	*y*	*z*	*U*_iso_*/*U*_eq_	
C1	−0.1391 (3)	−0.4315 (4)	−0.0093 (5)	0.0399 (11)	
C2	−0.0889 (3)	−0.4861 (5)	0.1175 (5)	0.0475 (12)	
H2	−0.0645	−0.4313	0.1955	0.057*	
C3	−0.0749 (3)	−0.6222 (5)	0.1283 (5)	0.0506 (13)	
H3	−0.0400	−0.6591	0.2129	0.061*	
C4	−0.1128 (3)	−0.7031 (4)	0.0134 (5)	0.0494 (13)	
C5	−0.1615 (3)	−0.6477 (5)	−0.1149 (5)	0.0612 (15)	
H5	−0.1848	−0.7023	−0.1936	0.073*	
C6	−0.1751 (3)	−0.5130 (5)	−0.1255 (4)	0.0572 (14)	
H6	−0.2087	−0.4759	−0.2110	0.069*	
C7	−0.4066 (4)	−0.3017 (4)	−0.0120 (5)	0.0566 (14)	
H7	−0.4032	−0.2745	0.0814	0.068*	
C8	−0.4945 (4)	−0.3538 (5)	−0.1000 (6)	0.0631 (15)	
C9	−0.4990 (4)	−0.4207 (5)	−0.2256 (6)	0.0806 (18)	
H9	−0.4455	−0.4349	−0.2562	0.097*	
C10	−0.5837 (5)	−0.4678 (5)	−0.3082 (7)	0.0977 (18)	
H10	−0.5875	−0.5122	−0.3942	0.117*	
C11	−0.6607 (5)	−0.4463 (6)	−0.2579 (7)	0.0966 (18)	
H11	−0.7171	−0.4770	−0.3127	0.116*	
C12	−0.6596 (5)	−0.3852 (6)	−0.1370 (7)	0.0965 (18)	
H12	−0.7135	−0.3750	−0.1062	0.116*	
C13	−0.5748 (4)	−0.3354 (5)	−0.0552 (6)	0.0836 (17)	
H13	−0.5728	−0.2898	0.0295	0.100*	
N1	−0.2581 (3)	−0.2379 (4)	0.0314 (3)	0.0489 (10)	
H1N	−0.254 (3)	−0.240 (4)	0.119 (2)	0.059*	
N2	−0.3361 (3)	−0.2933 (3)	−0.0607 (4)	0.0484 (10)	
O1	−0.1806 (2)	−0.2192 (3)	−0.1656 (3)	0.0587 (9)	
O2	−0.0928 (2)	−0.1919 (3)	0.0868 (3)	0.0574 (9)	
Cl1	−0.09997 (10)	−0.87458 (12)	0.02899 (14)	0.0712 (5)	
S1	−0.16184 (9)	−0.25981 (12)	−0.01912 (12)	0.0476 (4)	

### (E)-N'-Benzylidene-4-chlorobenzenesulfonohydrazide (I) Atomic displacement parameters (Å^2^)

**Table:**

	*U*^11^	*U*^22^	*U*^33^	*U*^12^	*U*^13^	*U*^23^
C1	0.035 (3)	0.046 (3)	0.034 (3)	0.000 (2)	0.002 (2)	−0.001 (2)
C2	0.040 (3)	0.051 (4)	0.045 (3)	−0.001 (3)	−0.001 (2)	−0.001 (3)
C3	0.042 (3)	0.058 (4)	0.044 (3)	0.002 (3)	−0.006 (2)	0.007 (3)
C4	0.044 (3)	0.053 (3)	0.053 (3)	0.004 (3)	0.017 (3)	0.001 (3)
C5	0.074 (4)	0.058 (4)	0.044 (3)	−0.007 (3)	0.000 (3)	−0.010 (3)
C6	0.068 (4)	0.051 (4)	0.041 (3)	0.006 (3)	−0.008 (3)	0.004 (3)
C7	0.048 (4)	0.054 (3)	0.062 (3)	0.001 (3)	0.003 (3)	0.003 (3)
C8	0.049 (4)	0.046 (3)	0.087 (4)	0.002 (3)	0.004 (3)	0.013 (3)
C9	0.074 (4)	0.065 (3)	0.091 (3)	−0.013 (3)	−0.003 (3)	0.001 (3)
C10	0.099 (4)	0.083 (3)	0.101 (3)	−0.016 (3)	0.007 (3)	−0.002 (3)
C11	0.082 (3)	0.081 (4)	0.113 (3)	−0.014 (3)	−0.002 (3)	0.017 (3)
C12	0.070 (3)	0.093 (4)	0.121 (4)	−0.004 (3)	0.013 (3)	0.016 (3)
C13	0.073 (4)	0.068 (4)	0.103 (4)	0.004 (3)	0.010 (3)	0.015 (3)
N1	0.044 (2)	0.060 (3)	0.037 (2)	−0.001 (2)	−0.001 (2)	−0.006 (2)
N2	0.041 (3)	0.046 (3)	0.049 (2)	−0.003 (2)	−0.005 (2)	−0.0005 (19)
O1	0.073 (2)	0.066 (2)	0.0337 (17)	−0.0022 (18)	0.0068 (15)	0.0120 (16)
O2	0.057 (2)	0.055 (2)	0.0502 (19)	−0.0147 (17)	−0.0068 (17)	−0.0039 (15)
Cl1	0.0744 (11)	0.0529 (9)	0.0819 (10)	0.0069 (8)	0.0112 (8)	0.0037 (7)
S1	0.0490 (8)	0.0505 (8)	0.0378 (7)	−0.0037 (7)	0.0001 (6)	0.0014 (7)

### (E)-N'-Benzylidene-4-chlorobenzenesulfonohydrazide (I) Geometric parameters (Å, º)

**Table:**

C1—C2	1.380 (5)	C8—C13	1.385 (7)
C1—C6	1.383 (5)	C9—C10	1.400 (7)
C1—S1	1.752 (4)	C9—H9	0.9300
C2—C3	1.379 (5)	C10—C11	1.372 (7)
C2—H2	0.9300	C10—H10	0.9300
C3—C4	1.377 (5)	C11—C12	1.313 (7)
C3—H3	0.9300	C11—H11	0.9300
C4—C5	1.387 (6)	C12—C13	1.409 (7)
C4—Cl1	1.732 (5)	C12—H12	0.9300
C5—C6	1.365 (6)	C13—H13	0.9300
C5—H5	0.9300	N1—N2	1.394 (5)
C6—H6	0.9300	N1—S1	1.644 (4)
C7—N2	1.258 (5)	N1—H1N	0.831 (18)
C7—C8	1.473 (6)	O1—S1	1.429 (3)
C7—H7	0.9300	O2—S1	1.432 (3)
C8—C9	1.371 (6)		
			
C2—C1—C6	120.1 (4)	C8—C9—H9	119.7
C2—C1—S1	119.5 (4)	C10—C9—H9	119.7
C6—C1—S1	120.3 (4)	C11—C10—C9	117.7 (6)
C3—C2—C1	119.8 (4)	C11—C10—H10	121.1
C3—C2—H2	120.1	C9—C10—H10	121.1
C1—C2—H2	120.1	C12—C11—C10	124.0 (7)
C4—C3—C2	119.8 (4)	C12—C11—H11	118.0
C4—C3—H3	120.1	C10—C11—H11	118.0
C2—C3—H3	120.1	C11—C12—C13	118.3 (7)
C3—C4—C5	120.3 (4)	C11—C12—H12	120.9
C3—C4—Cl1	120.0 (4)	C13—C12—H12	120.9
C5—C4—Cl1	119.8 (4)	C8—C13—C12	120.5 (6)
C6—C5—C4	119.8 (4)	C8—C13—H13	119.7
C6—C5—H5	120.1	C12—C13—H13	119.7
C4—C5—H5	120.1	N2—N1—S1	114.5 (3)
C5—C6—C1	120.1 (4)	N2—N1—H1N	118 (3)
C5—C6—H6	119.9	S1—N1—H1N	117 (3)
C1—C6—H6	119.9	C7—N2—N1	115.8 (4)
N2—C7—C8	121.3 (5)	O1—S1—O2	120.03 (19)
N2—C7—H7	119.4	O1—S1—N1	106.33 (18)
C8—C7—H7	119.4	O2—S1—N1	104.79 (18)
C9—C8—C13	118.9 (6)	O1—S1—C1	108.75 (19)
C9—C8—C7	121.7 (5)	O2—S1—C1	109.4 (2)
C13—C8—C7	119.5 (6)	N1—S1—C1	106.7 (2)
C8—C9—C10	120.5 (6)		
			
C6—C1—C2—C3	−0.3 (7)	C10—C11—C12—C13	1.6 (9)
S1—C1—C2—C3	176.6 (3)	C9—C8—C13—C12	0.4 (8)
C1—C2—C3—C4	−1.4 (6)	C7—C8—C13—C12	−179.5 (5)
C2—C3—C4—C5	2.8 (7)	C11—C12—C13—C8	−1.6 (9)
C2—C3—C4—Cl1	−177.1 (3)	C8—C7—N2—N1	177.8 (4)
C3—C4—C5—C6	−2.7 (7)	S1—N1—N2—C7	166.5 (3)
Cl1—C4—C5—C6	177.2 (4)	N2—N1—S1—O1	49.9 (3)
C4—C5—C6—C1	1.1 (7)	N2—N1—S1—O2	178.0 (3)
C2—C1—C6—C5	0.4 (7)	N2—N1—S1—C1	−66.0 (3)
S1—C1—C6—C5	−176.4 (4)	C2—C1—S1—O1	157.4 (3)
N2—C7—C8—C9	13.9 (7)	C6—C1—S1—O1	−25.7 (4)
N2—C7—C8—C13	−166.2 (5)	C2—C1—S1—O2	24.6 (4)
C13—C8—C9—C10	0.9 (8)	C6—C1—S1—O2	−158.6 (3)
C7—C8—C9—C10	−179.2 (5)	C2—C1—S1—N1	−88.2 (4)
C8—C9—C10—C11	−0.9 (8)	C6—C1—S1—N1	88.6 (4)
C9—C10—C11—C12	−0.4 (9)		

### (E)-N'-Benzylidene-4-chlorobenzenesulfonohydrazide (I) Hydrogen-bond geometry (Å, º)

**Table:**

*D*—H···*A*	*D*—H	H···*A*	*D*···*A*	*D*—H···*A*
N1—H1*N*···O1^i^	0.83 (2)	2.14 (3)	2.897 (4)	152 (4)
C3—H3···O2^ii^	0.93	2.43	3.305 (5)	158

Symmetry codes: (i) *x*, −*y*−1/2, *z*+1/2; (ii) −*x*, *y*−1/2, −*z*+1/2.

### (E)-4-Chloro-N'-(2-methylbenzylidene)benzenesulfonohydrazide (II) Crystal data

**Table:**

C_14_H_13_ClN_2_O_2_S	*F*(000) = 640
*M**_r_* = 308.77	*D*_x_ = 1.423 Mg m^−^^3^
Monoclinic, *P*2_1_/*c*	Mo *K*α radiation, λ = 0.71073 Å
*a* = 15.034 (2) Å	Cell parameters from 1546 reflections
*b* = 10.180 (1) Å	θ = 2.8–27.7°
*c* = 9.8119 (9) Å	µ = 0.41 mm^−^^1^
β = 106.34 (1)°	*T* = 293 K
*V* = 1441.0 (3) Å^3^	Rod, colourless
*Z* = 4	0.22 × 0.16 × 0.08 mm

### (E)-4-Chloro-N'-(2-methylbenzylidene)benzenesulfonohydrazide (II) Data collection

**Table:**

Oxford Diffraction Xcalibur diffractometer with Sapphire CCD detector	1713 reflections with *I* > 2σ(*I*)
Radiation source: Enhance (Mo) X-ray Source	*R*_int_ = 0.038
Rotation method data acquisition using ω scans.	θ_max_ = 25.4°, θ_min_ = 2.8°
Absorption correction: multi-scan (CrysAlis RED; Oxford Diffraction, 2009)	*h* = −13→18
*T*_min_ = 0.915, *T*_max_ = 0.968	*k* = −9→12
5157 measured reflections	*l* = −11→11
2636 independent reflections	

### (E)-4-Chloro-N'-(2-methylbenzylidene)benzenesulfonohydrazide (II) Refinement

**Table:**

Refinement on *F*^2^	32 restraints
Least-squares matrix: full	Hydrogen site location: mixed
*R*[*F*^2^ > 2σ(*F*^2^)] = 0.067	H atoms treated by a mixture of independent and constrained refinement
*wR*(*F*^2^) = 0.195	*w* = 1/[σ^2^(*F*_o_^2^) + (0.0912*P*)^2^ + 1.1282*P*] where *P* = (*F*_o_^2^ + 2*F*_c_^2^)/3
*S* = 1.07	(Δ/σ)_max_ < 0.001
2636 reflections	Δρ_max_ = 0.66 e Å^−^^3^
185 parameters	Δρ_min_ = −0.32 e Å^−^^3^

### (E)-4-Chloro-N'-(2-methylbenzylidene)benzenesulfonohydrazide (II) Special details

**Table:**

Geometry. All esds (except the esd in the dihedral angle between two l.s. planes) are estimated using the full covariance matrix. The cell esds are taken into account individually in the estimation of esds in distances, angles and torsion angles; correlations between esds in cell parameters are only used when they are defined by crystal symmetry. An approximate (isotropic) treatment of cell esds is used for estimating esds involving l.s. planes.

### (E)-4-Chloro-N'-(2-methylbenzylidene)benzenesulfonohydrazide (II) Fractional atomic coordinates and isotropic or equivalent isotropic displacement parameters (Å^2^)

**Table:**

	*x*	*y*	*z*	*U*_iso_*/*U*_eq_	
C1	0.1380 (3)	0.5693 (5)	0.0113 (4)	0.0511 (11)	
C2	0.1696 (3)	0.4872 (5)	0.1299 (4)	0.0603 (12)	
H2	0.2001	0.5234	0.2176	0.072*	
C3	0.1560 (3)	0.3542 (5)	0.1178 (5)	0.0637 (13)	
H3	0.1783	0.2995	0.1959	0.076*	
C4	0.1083 (3)	0.3027 (5)	−0.0130 (5)	0.0592 (12)	
C5	0.0748 (3)	0.3818 (5)	−0.1302 (5)	0.0604 (12)	
H5	0.0424	0.3454	−0.2168	0.072*	
C6	0.0896 (3)	0.5142 (5)	−0.1179 (4)	0.0586 (12)	
H6	0.0671	0.5679	−0.1967	0.070*	
C7	0.4065 (3)	0.6873 (4)	0.0171 (5)	0.0568 (11)	
H7	0.3990	0.7111	−0.0770	0.068*	
C8	0.4969 (3)	0.6382 (5)	0.0999 (6)	0.0679 (11)	
C9	0.5125 (4)	0.5615 (5)	0.2183 (6)	0.0810 (12)	
C10	0.6096 (4)	0.5259 (6)	0.2885 (7)	0.0973 (16)	
H10	0.6255	0.4756	0.3710	0.117*	
C11	0.6757 (4)	0.5696 (7)	0.2276 (8)	0.1023 (18)	
H11	0.7370	0.5475	0.2724	0.123*	
C12	0.6595 (5)	0.6409 (7)	0.1098 (9)	0.109 (2)	
H12	0.7078	0.6654	0.0733	0.130*	
C13	0.5728 (4)	0.6766 (6)	0.0452 (7)	0.0871 (15)	
H13	0.5608	0.7274	−0.0368	0.104*	
C14	0.4420 (5)	0.5137 (7)	0.2741 (6)	0.0985 (19)	
H14A	0.4687	0.4582	0.3543	0.148*	
H14B	0.3981	0.4642	0.2025	0.148*	
H14C	0.4110	0.5863	0.3036	0.148*	
N1	0.2586 (2)	0.7565 (4)	−0.0264 (3)	0.0539 (9)	
H1N	0.245 (3)	0.748 (4)	−0.118 (2)	0.065*	
N2	0.3375 (2)	0.6990 (4)	0.0672 (3)	0.0539 (9)	
O1	0.0942 (2)	0.8064 (3)	−0.0839 (3)	0.0690 (9)	
O2	0.1843 (2)	0.7771 (3)	0.1677 (3)	0.0685 (9)	
Cl1	0.09551 (9)	0.13260 (14)	−0.02983 (15)	0.0787 (5)	
S1	0.16318 (7)	0.73763 (12)	0.02214 (10)	0.0551 (4)	

### (E)-4-Chloro-N'-(2-methylbenzylidene)benzenesulfonohydrazide (II) Atomic displacement parameters (Å^2^)

**Table:**

	*U*^11^	*U*^22^	*U*^33^	*U*^12^	*U*^13^	*U*^23^
C1	0.041 (2)	0.075 (3)	0.037 (2)	0.000 (2)	0.0101 (16)	−0.004 (2)
C2	0.060 (3)	0.077 (4)	0.041 (2)	−0.005 (2)	0.0085 (19)	0.000 (2)
C3	0.066 (3)	0.072 (4)	0.051 (3)	−0.002 (2)	0.013 (2)	0.006 (2)
C4	0.043 (2)	0.081 (3)	0.056 (3)	−0.004 (2)	0.0170 (19)	−0.005 (2)
C5	0.046 (2)	0.082 (4)	0.047 (2)	−0.005 (2)	0.0034 (19)	−0.007 (2)
C6	0.044 (2)	0.085 (4)	0.041 (2)	0.006 (2)	0.0023 (17)	0.005 (2)
C7	0.058 (3)	0.052 (3)	0.058 (3)	0.003 (2)	0.013 (2)	0.003 (2)
C8	0.062 (2)	0.049 (3)	0.087 (3)	0.001 (2)	0.012 (2)	−0.0128 (18)
C9	0.088 (2)	0.062 (3)	0.082 (3)	0.006 (3)	0.006 (2)	−0.011 (2)
C10	0.096 (3)	0.084 (3)	0.099 (3)	0.020 (3)	0.006 (2)	0.001 (3)
C11	0.085 (2)	0.095 (4)	0.112 (4)	0.007 (3)	0.004 (3)	−0.012 (3)
C12	0.089 (4)	0.104 (4)	0.131 (4)	−0.001 (3)	0.027 (3)	−0.018 (3)
C13	0.066 (3)	0.083 (3)	0.112 (3)	0.000 (2)	0.025 (2)	−0.016 (3)
C14	0.123 (5)	0.087 (4)	0.079 (4)	0.020 (4)	0.019 (4)	0.012 (3)
N1	0.053 (2)	0.066 (2)	0.0376 (17)	0.0006 (17)	0.0055 (15)	0.0026 (18)
N2	0.049 (2)	0.061 (2)	0.0474 (19)	0.0036 (17)	0.0057 (16)	0.0021 (17)
O1	0.0607 (19)	0.081 (2)	0.0580 (18)	0.0250 (17)	0.0050 (15)	0.0039 (17)
O2	0.080 (2)	0.083 (2)	0.0421 (16)	0.0000 (17)	0.0162 (15)	−0.0115 (15)
Cl1	0.0758 (9)	0.0776 (9)	0.0815 (9)	−0.0119 (7)	0.0200 (7)	−0.0071 (7)
S1	0.0512 (6)	0.0729 (8)	0.0384 (6)	0.0101 (5)	0.0081 (4)	−0.0012 (5)

### (E)-4-Chloro-N'-(2-methylbenzylidene)benzenesulfonohydrazide (II) Geometric parameters (Å, º)

**Table:**

C1—C6	1.390 (6)	C9—C14	1.409 (8)
C1—C2	1.403 (6)	C9—C10	1.474 (8)
C1—S1	1.751 (5)	C10—C11	1.369 (9)
C2—C3	1.369 (7)	C10—H10	0.9300
C2—H2	0.9300	C11—C12	1.328 (9)
C3—C4	1.385 (6)	C11—H11	0.9300
C3—H3	0.9300	C12—C13	1.329 (8)
C4—C5	1.378 (6)	C12—H12	0.9300
C4—Cl1	1.745 (5)	C13—H13	0.9300
C5—C6	1.365 (7)	C14—H14A	0.9600
C5—H5	0.9300	C14—H14B	0.9600
C6—H6	0.9300	C14—H14C	0.9600
C7—N2	1.272 (5)	N1—N2	1.407 (5)
C7—C8	1.461 (6)	N1—S1	1.645 (4)
C7—H7	0.9300	N1—H1N	0.864 (19)
C8—C9	1.365 (7)	O1—S1	1.428 (3)
C8—C13	1.446 (8)	O2—S1	1.431 (3)
			
C6—C1—C2	118.9 (5)	C11—C10—H10	121.3
C6—C1—S1	119.9 (3)	C9—C10—H10	121.3
C2—C1—S1	121.1 (3)	C12—C11—C10	125.4 (7)
C3—C2—C1	120.7 (4)	C12—C11—H11	117.3
C3—C2—H2	119.7	C10—C11—H11	117.3
C1—C2—H2	119.7	C13—C12—C11	118.7 (7)
C2—C3—C4	118.7 (5)	C13—C12—H12	120.7
C2—C3—H3	120.6	C11—C12—H12	120.7
C4—C3—H3	120.6	C12—C13—C8	121.3 (7)
C5—C4—C3	121.6 (5)	C12—C13—H13	119.3
C5—C4—Cl1	119.8 (4)	C8—C13—H13	119.3
C3—C4—Cl1	118.6 (4)	C9—C14—H14A	109.5
C6—C5—C4	119.4 (4)	C9—C14—H14B	109.5
C6—C5—H5	120.3	H14A—C14—H14B	109.5
C4—C5—H5	120.3	C9—C14—H14C	109.5
C5—C6—C1	120.6 (4)	H14A—C14—H14C	109.5
C5—C6—H6	119.7	H14B—C14—H14C	109.5
C1—C6—H6	119.7	N2—N1—S1	114.0 (3)
N2—C7—C8	123.3 (4)	N2—N1—H1N	123 (3)
N2—C7—H7	118.3	S1—N1—H1N	108 (3)
C8—C7—H7	118.3	C7—N2—N1	114.6 (4)
C9—C8—C13	120.4 (5)	O1—S1—O2	120.1 (2)
C9—C8—C7	125.4 (5)	O1—S1—N1	104.20 (19)
C13—C8—C7	114.2 (5)	O2—S1—N1	106.83 (19)
C8—C9—C14	124.1 (5)	O1—S1—C1	109.6 (2)
C8—C9—C10	116.7 (6)	O2—S1—C1	108.58 (19)
C14—C9—C10	119.2 (6)	N1—S1—C1	106.73 (19)
C11—C10—C9	117.5 (6)		
			
C6—C1—C2—C3	2.3 (6)	C14—C9—C10—C11	177.3 (6)
S1—C1—C2—C3	−174.8 (4)	C9—C10—C11—C12	−0.4 (10)
C1—C2—C3—C4	−1.6 (7)	C10—C11—C12—C13	1.5 (11)
C2—C3—C4—C5	0.1 (7)	C11—C12—C13—C8	−0.6 (10)
C2—C3—C4—Cl1	177.3 (4)	C9—C8—C13—C12	−1.4 (8)
C3—C4—C5—C6	0.7 (7)	C7—C8—C13—C12	179.2 (5)
Cl1—C4—C5—C6	−176.4 (3)	C8—C7—N2—N1	175.8 (4)
C4—C5—C6—C1	0.0 (7)	S1—N1—N2—C7	165.4 (3)
C2—C1—C6—C5	−1.5 (6)	N2—N1—S1—O1	178.1 (3)
S1—C1—C6—C5	175.6 (3)	N2—N1—S1—O2	50.0 (3)
N2—C7—C8—C9	22.4 (7)	N2—N1—S1—C1	−66.0 (3)
N2—C7—C8—C13	−158.3 (5)	C6—C1—S1—O1	28.2 (4)
C13—C8—C9—C14	−176.4 (5)	C2—C1—S1—O1	−154.8 (3)
C7—C8—C9—C14	2.9 (8)	C6—C1—S1—O2	161.1 (3)
C13—C8—C9—C10	2.4 (7)	C2—C1—S1—O2	−21.9 (4)
C7—C8—C9—C10	−178.3 (5)	C6—C1—S1—N1	−84.1 (3)
C8—C9—C10—C11	−1.6 (8)	C2—C1—S1—N1	92.9 (4)

### (E)-4-Chloro-N'-(2-methylbenzylidene)benzenesulfonohydrazide (II) Hydrogen-bond geometry (Å, º)

**Table:**

*D*—H···*A*	*D*—H	H···*A*	*D*···*A*	*D*—H···*A*
N1—H1*N*···O2^i^	0.86 (2)	2.06 (2)	2.913 (4)	168 (4)
C5—H5···O1^ii^	0.93	2.44	3.303 (5)	155

Symmetry codes: (i) *x*, −*y*+3/2, *z*−1/2; (ii) −*x*, *y*−1/2, −*z*−1/2.

### (E)-4-Chloro-N'-(4-methylbenzylidene)benzenesulfonohydrazide (III) Crystal data

**Table:**

C_14_H_13_ClN_2_O_2_S	*F*(000) = 640
*M**_r_* = 308.77	*D*_x_ = 1.407 Mg m^−^^3^
Monoclinic, *P*2_1_/*n*	Mo *K*α radiation, λ = 0.71073 Å
*a* = 9.406 (1) Å	Cell parameters from 3029 reflections
*b* = 5.8353 (6) Å	θ = 2.9–27.8°
*c* = 26.930 (2) Å	µ = 0.41 mm^−^^1^
β = 99.621 (9)°	*T* = 293 K
*V* = 1457.3 (2) Å^3^	Rod, colourless
*Z* = 4	0.48 × 0.16 × 0.14 mm

### (E)-4-Chloro-N'-(4-methylbenzylidene)benzenesulfonohydrazide (III) Data collection

**Table:**

Oxford Diffraction Xcalibur diffractometer with Sapphire CCD detector	2106 reflections with *I* > 2σ(*I*)
Radiation source: Enhance (Mo) X-ray Source	*R*_int_ = 0.027
Rotation method data acquisition using ω scans.	θ_max_ = 25.4°, θ_min_ = 3.1°
Absorption correction: multi-scan (CrysAlis RED; Oxford Diffraction, 2009)	*h* = −11→11
*T*_min_ = 0.829, *T*_max_ = 0.945	*k* = −7→7
9653 measured reflections	*l* = −31→32
2652 independent reflections	

### (E)-4-Chloro-N'-(4-methylbenzylidene)benzenesulfonohydrazide (III) Refinement

**Table:**

Refinement on *F*^2^	1 restraint
Least-squares matrix: full	Hydrogen site location: mixed
*R*[*F*^2^ > 2σ(*F*^2^)] = 0.040	H atoms treated by a mixture of independent and constrained refinement
*wR*(*F*^2^) = 0.095	*w* = 1/[σ^2^(*F*_o_^2^) + (0.0352*P*)^2^ + 0.7905*P*] where *P* = (*F*_o_^2^ + 2*F*_c_^2^)/3
*S* = 1.05	(Δ/σ)_max_ = 0.001
2652 reflections	Δρ_max_ = 0.21 e Å^−^^3^
185 parameters	Δρ_min_ = −0.30 e Å^−^^3^

### (E)-4-Chloro-N'-(4-methylbenzylidene)benzenesulfonohydrazide (III) Special details

**Table:**

Geometry. All esds (except the esd in the dihedral angle between two l.s. planes) are estimated using the full covariance matrix. The cell esds are taken into account individually in the estimation of esds in distances, angles and torsion angles; correlations between esds in cell parameters are only used when they are defined by crystal symmetry. An approximate (isotropic) treatment of cell esds is used for estimating esds involving l.s. planes.

### (E)-4-Chloro-N'-(4-methylbenzylidene)benzenesulfonohydrazide (III) Fractional atomic coordinates and isotropic or equivalent isotropic displacement parameters (Å^2^)

**Table:**

	*x*	*y*	*z*	*U*_iso_*/*U*_eq_	
Cl1	1.21023 (8)	0.70656 (16)	0.14097 (3)	0.0843 (3)	
S1	0.69509 (6)	0.24496 (9)	0.00160 (2)	0.03860 (16)	
O1	0.62933 (17)	0.4035 (3)	−0.03599 (5)	0.0493 (4)	
O2	0.74225 (17)	0.0275 (3)	−0.01324 (6)	0.0501 (4)	
N1	0.5723 (2)	0.2120 (3)	0.03686 (7)	0.0445 (5)	
H1N	0.517 (2)	0.326 (3)	0.0367 (9)	0.053*	
N2	0.60598 (19)	0.0841 (3)	0.08093 (7)	0.0434 (4)	
C1	0.8426 (2)	0.3768 (4)	0.03983 (7)	0.0361 (5)	
C2	0.8228 (3)	0.5883 (4)	0.06121 (8)	0.0464 (6)	
H2	0.7336	0.6608	0.0546	0.056*	
C3	0.9362 (3)	0.6891 (4)	0.09212 (9)	0.0537 (6)	
H3	0.9244	0.8305	0.1069	0.064*	
C4	1.0678 (3)	0.5796 (4)	0.10118 (8)	0.0511 (6)	
C5	1.0885 (2)	0.3719 (4)	0.07935 (9)	0.0523 (6)	
H5	1.1783	0.3014	0.0853	0.063*	
C6	0.9746 (2)	0.2696 (4)	0.04858 (8)	0.0441 (5)	
H6	0.9869	0.1286	0.0338	0.053*	
C7	0.5267 (2)	0.1280 (4)	0.11369 (8)	0.0457 (6)	
H7	0.4591	0.2450	0.1070	0.055*	
C8	0.5363 (2)	0.0042 (4)	0.16116 (8)	0.0447 (5)	
C9	0.6130 (3)	−0.1985 (4)	0.17103 (10)	0.0557 (7)	
H9	0.6628	−0.2591	0.1470	0.067*	
C10	0.6159 (3)	−0.3103 (5)	0.21616 (10)	0.0654 (7)	
H10	0.6690	−0.4448	0.2223	0.078*	
C11	0.5422 (3)	−0.2279 (5)	0.25255 (10)	0.0614 (7)	
C12	0.4659 (3)	−0.0281 (5)	0.24265 (10)	0.0653 (8)	
H12	0.4155	0.0308	0.2667	0.078*	
C13	0.4623 (3)	0.0876 (5)	0.19761 (9)	0.0572 (7)	
H13	0.4096	0.2226	0.1918	0.069*	
C14	0.5452 (4)	−0.3541 (7)	0.30183 (11)	0.0970 (11)	
H14A	0.4539	−0.3369	0.3129	0.146*	
H14B	0.6201	−0.2918	0.3268	0.146*	
H14C	0.5636	−0.5138	0.2971	0.146*	

### (E)-4-Chloro-N'-(4-methylbenzylidene)benzenesulfonohydrazide (III) Atomic displacement parameters (Å^2^)

**Table:**

	*U*^11^	*U*^22^	*U*^33^	*U*^12^	*U*^13^	*U*^23^
Cl1	0.0677 (5)	0.1156 (7)	0.0697 (5)	−0.0376 (5)	0.0119 (4)	−0.0303 (5)
S1	0.0379 (3)	0.0396 (3)	0.0393 (3)	0.0037 (3)	0.0093 (2)	−0.0012 (3)
O1	0.0511 (9)	0.0574 (10)	0.0403 (8)	0.0112 (8)	0.0099 (7)	0.0085 (8)
O2	0.0505 (9)	0.0428 (9)	0.0572 (10)	0.0034 (7)	0.0097 (7)	−0.0130 (8)
N1	0.0398 (10)	0.0472 (12)	0.0483 (10)	0.0086 (9)	0.0129 (8)	0.0081 (10)
N2	0.0406 (10)	0.0415 (10)	0.0485 (10)	−0.0008 (8)	0.0086 (9)	0.0067 (9)
C1	0.0388 (12)	0.0348 (11)	0.0370 (11)	0.0014 (9)	0.0129 (9)	0.0011 (9)
C2	0.0519 (14)	0.0378 (12)	0.0504 (13)	0.0075 (11)	0.0109 (11)	−0.0011 (11)
C3	0.0678 (17)	0.0410 (13)	0.0547 (14)	−0.0065 (12)	0.0173 (13)	−0.0093 (12)
C4	0.0499 (14)	0.0629 (16)	0.0423 (12)	−0.0185 (12)	0.0131 (11)	−0.0075 (12)
C5	0.0368 (13)	0.0638 (16)	0.0569 (14)	0.0016 (12)	0.0099 (11)	−0.0040 (13)
C6	0.0410 (12)	0.0433 (13)	0.0504 (13)	0.0021 (11)	0.0146 (10)	−0.0066 (11)
C7	0.0381 (12)	0.0483 (14)	0.0507 (13)	0.0032 (11)	0.0071 (10)	0.0029 (11)
C8	0.0408 (12)	0.0482 (13)	0.0455 (13)	−0.0054 (11)	0.0089 (10)	0.0015 (11)
C9	0.0562 (15)	0.0554 (16)	0.0595 (15)	0.0073 (12)	0.0210 (12)	0.0073 (13)
C10	0.0693 (18)	0.0591 (17)	0.0704 (17)	0.0118 (14)	0.0197 (14)	0.0191 (14)
C11	0.0667 (17)	0.0654 (17)	0.0535 (15)	−0.0035 (14)	0.0138 (13)	0.0098 (14)
C12	0.079 (2)	0.0709 (19)	0.0507 (15)	0.0037 (16)	0.0239 (14)	−0.0005 (14)
C13	0.0621 (16)	0.0549 (15)	0.0565 (15)	0.0074 (13)	0.0156 (12)	0.0029 (13)
C14	0.126 (3)	0.105 (3)	0.0663 (19)	0.014 (2)	0.0326 (19)	0.032 (2)

### (E)-4-Chloro-N'-(4-methylbenzylidene)benzenesulfonohydrazide (III) Geometric parameters (Å, º)

**Table:**

Cl1—C4	1.735 (2)	C6—H6	0.9300
S1—O2	1.4232 (16)	C7—C8	1.458 (3)
S1—O1	1.4330 (15)	C7—H7	0.9300
S1—N1	1.6248 (18)	C8—C13	1.383 (3)
S1—C1	1.761 (2)	C8—C9	1.388 (3)
N1—N2	1.393 (2)	C9—C10	1.376 (3)
N1—H1N	0.845 (16)	C9—H9	0.9300
N2—C7	1.273 (3)	C10—C11	1.378 (4)
C1—C6	1.375 (3)	C10—H10	0.9300
C1—C2	1.387 (3)	C11—C12	1.372 (4)
C2—C3	1.371 (3)	C11—C14	1.514 (4)
C2—H2	0.9300	C12—C13	1.383 (3)
C3—C4	1.378 (3)	C12—H12	0.9300
C3—H3	0.9300	C13—H13	0.9300
C4—C5	1.375 (3)	C14—H14A	0.9600
C5—C6	1.376 (3)	C14—H14B	0.9600
C5—H5	0.9300	C14—H14C	0.9600
			
O2—S1—O1	119.69 (9)	N2—C7—C8	123.4 (2)
O2—S1—N1	110.11 (10)	N2—C7—H7	118.3
O1—S1—N1	102.95 (9)	C8—C7—H7	118.3
O2—S1—C1	107.55 (10)	C13—C8—C9	118.1 (2)
O1—S1—C1	109.63 (10)	C13—C8—C7	118.9 (2)
N1—S1—C1	106.13 (10)	C9—C8—C7	123.0 (2)
N2—N1—S1	118.59 (14)	C10—C9—C8	120.4 (2)
N2—N1—H1N	118.5 (16)	C10—C9—H9	119.8
S1—N1—H1N	113.8 (17)	C8—C9—H9	119.8
C7—N2—N1	113.99 (18)	C9—C10—C11	121.6 (3)
C6—C1—C2	120.9 (2)	C9—C10—H10	119.2
C6—C1—S1	120.14 (16)	C11—C10—H10	119.2
C2—C1—S1	118.94 (17)	C12—C11—C10	117.9 (2)
C3—C2—C1	119.3 (2)	C12—C11—C14	121.1 (3)
C3—C2—H2	120.4	C10—C11—C14	121.0 (3)
C1—C2—H2	120.4	C11—C12—C13	121.4 (3)
C2—C3—C4	119.5 (2)	C11—C12—H12	119.3
C2—C3—H3	120.2	C13—C12—H12	119.3
C4—C3—H3	120.2	C12—C13—C8	120.6 (3)
C5—C4—C3	121.3 (2)	C12—C13—H13	119.7
C5—C4—Cl1	119.4 (2)	C8—C13—H13	119.7
C3—C4—Cl1	119.3 (2)	C11—C14—H14A	109.5
C4—C5—C6	119.3 (2)	C11—C14—H14B	109.5
C4—C5—H5	120.4	H14A—C14—H14B	109.5
C6—C5—H5	120.4	C11—C14—H14C	109.5
C1—C6—C5	119.7 (2)	H14A—C14—H14C	109.5
C1—C6—H6	120.2	H14B—C14—H14C	109.5
C5—C6—H6	120.2		
			
O2—S1—N1—N2	57.73 (19)	C2—C1—C6—C5	0.6 (3)
O1—S1—N1—N2	−173.56 (16)	S1—C1—C6—C5	−178.67 (17)
C1—S1—N1—N2	−58.39 (18)	C4—C5—C6—C1	0.5 (3)
S1—N1—N2—C7	157.85 (17)	N1—N2—C7—C8	175.80 (19)
O2—S1—C1—C6	2.2 (2)	N2—C7—C8—C13	169.8 (2)
O1—S1—C1—C6	−129.39 (17)	N2—C7—C8—C9	−12.3 (4)
N1—S1—C1—C6	120.07 (18)	C13—C8—C9—C10	−0.8 (4)
O2—S1—C1—C2	−177.10 (16)	C7—C8—C9—C10	−178.7 (2)
O1—S1—C1—C2	51.28 (18)	C8—C9—C10—C11	0.9 (4)
N1—S1—C1—C2	−59.26 (18)	C9—C10—C11—C12	−0.6 (4)
C6—C1—C2—C3	−1.1 (3)	C9—C10—C11—C14	179.4 (3)
S1—C1—C2—C3	178.25 (17)	C10—C11—C12—C13	0.2 (4)
C1—C2—C3—C4	0.4 (3)	C14—C11—C12—C13	−179.8 (3)
C2—C3—C4—C5	0.8 (4)	C11—C12—C13—C8	−0.1 (4)
C2—C3—C4—Cl1	−179.19 (17)	C9—C8—C13—C12	0.4 (4)
C3—C4—C5—C6	−1.2 (4)	C7—C8—C13—C12	178.4 (2)
Cl1—C4—C5—C6	178.76 (18)		

### (E)-4-Chloro-N'-(4-methylbenzylidene)benzenesulfonohydrazide (III) Hydrogen-bond geometry (Å, º)

Cg1 is the centroid of ring C8-C13.

**Table:**

*D*—H···*A*	*D*—H	H···*A*	*D*···*A*	*D*—H···*A*
N1—H1*N*···O1^i^	0.85 (2)	2.09 (2)	2.935 (2)	177 (2)
C4—Cl1···*Cg*1^ii^	1.74 (1)	3.47 (1)	5.175 (3)	168 (1)

Symmetry codes: (i) −*x*+1, −*y*+1, −*z*; (ii) *x*+1, *y*+1, *z*.
